# Influence of posterior pedicle screw fixation at L4–L5 level on biomechanics of the lumbar spine with and without fusion: a finite element method

**DOI:** 10.1186/s12938-021-00940-1

**Published:** 2021-10-07

**Authors:** Emre Sengul, Ramazan Ozmen, Mesut Emre Yaman, Teyfik Demir

**Affiliations:** 1 Department of Mechanical Engineering, TOBB University of Economics and Technology, Çankaya, Ankara, Turkey; 2 Department of Mechanical Engineering, Karabük University, Merkez, Karabük, Turkey; 3 Department of Neurosurgery, Gazi University School of Medicine, Ankara, Turkey; 4 Mechanical Engineer, Roketsan Inc., Lalahan, 06852 Ankara, Turkey

**Keywords:** Biomechanics, Lumbar spine, Posterior pedicle screw, Finite element method

## Abstract

**Background:**

Posterior pedicle screw (PS) fixation, a common treatment method for widespread low-back pain problems, has many uncertain aspects including stress concentration levels, effects on adjacent segments, and relationships with physiological motions. A better understanding of how posterior PS fixation affects the biomechanics of the lumbar spine is needed. For this purpose, a finite element (FE) model of a lumbar spine with posterior PS fixation at the L4–L5 segment level was developed by partially removing facet joints (FJs) to imitate an actual surgical procedure. This FE study aimed to investigate the influence of the posterior PS fixation system on the biomechanics of the lumbar spine before and after fusion by determining which physiological motions have the most increase in posterior instrumentation (PI) stresses and FJ loading.

**Results:**

It was determined that posterior PS fixation increased FJ loading by approximately 35% and 23% at the L3–L4 adjacent level with extension and lateral bending motion, respectively. This increase in FJ loading at the adjacent level could point to the possibility that adjacent segment disease has developed or progressed after posterior lumbar interbody fusion. Furthermore, analyses of peak von Mises stresses on PI showed that the maximum PI stresses of 272.1 MPa and 263.7 MPa occurred in lateral bending and flexion motion before fusion, respectively.

**Conclusions:**

The effects of a posterior PS fixation system on the biomechanics of the lumbar spine before and after fusion were investigated for all physiological motions. This model could be used as a fundamental tool for further studies, providing a better understanding of the effects of posterior PS fixation by clearing up uncertain aspects.

## Background

Low-back pain is a commonly faced phenomenon due to spinal instabilities, deformities, and degenerative diseases [1]. In the treatment procedures for these problems, such as arthrodesis, the pedicle screw (PS) fixation method is widely used and regarded as a gold standard in fusion surgery [2–4]. PS fixation is usually applied to generate fusion at the implant level for treatment. It has many assets, such as a high fusion rate, maintenance of the original disc height, and maintenance of lumbar spine stability [5, 6]. Nevertheless, the PS fixation method with interbody fusion has some complications including PS failure, rod breakage, and adjacent segment disease (ASD) as a result of restricting the range of motion (ROM) of the lumbar spine [7–10]. Many experimental and numerical studies have been conducted based on fusion surgery to benefit from these advantages more efficiently and avoid the disadvantages [11–14]. FE analysis offers more advantages over cadaver experimental studies in terms of lower cost, higher efficiency, and estimation of internal stress in bones and on spinal implants in spite of the disadvantages of validation requirement [4]. In addition, the FE method has made it easier to properly investigate the stabilization of degenerated spines, allowing the consideration of rigid and dynamic fixation or the effects of different rod and cage materials and locations. In the study of Xu et al. [4], the authors evaluated rod stress under different loading conditions, and they stated that using longer rods with intermediate screws gives extra support for the fixation device, but more screws mean more stress concentrated on the rods, while the interbody cage slightly reduces the load on the posterior fixation devices and anterior support. Some studies then showed that this rigid fixation can cause abnormal changes in the load transfer of the spine, which can cause stresses leading to degeneration of intervertebral discs and bony structures [15]. Therefore, some FE studies have investigated different rod materials for posterior PS fixation to overcome this problem [15–17].

As mentioned above, PS fixation and interbody fusion restricts the ROM of the lumbar spine; therefore, it might cause an increase in ASD. However, no certain conclusions have been reached regarding the biomechanics or risk factors at play in the relationship between ASD and lumbar fusion surgery [18–21]. Non-fusion dynamic methods of PS fixation were developed to eliminate the possible disadvantages of fusion achieved with PS fixation [22–24]. Although these approaches provided some advantages such as reducing surgical morbidity and cases of ASD, they also introduced some problems of screw loosening due to the dynamic structure [11, 25, 26]. For both fusion and non-fusion methods, posterior PS fixation is fundamental. Therefore, the most important thing to pursue is a better understanding of the effects of posterior PS fixation on the biomechanics of the lumbar spine. This study aimed to investigate the influence of a posterior PS fixation system on the biomechanics of the lumbar spine before and after fusion by determining changes in PI stresses and FJ loading under different physiological motions.

## Results

### Model validation

**Fig. 1:**
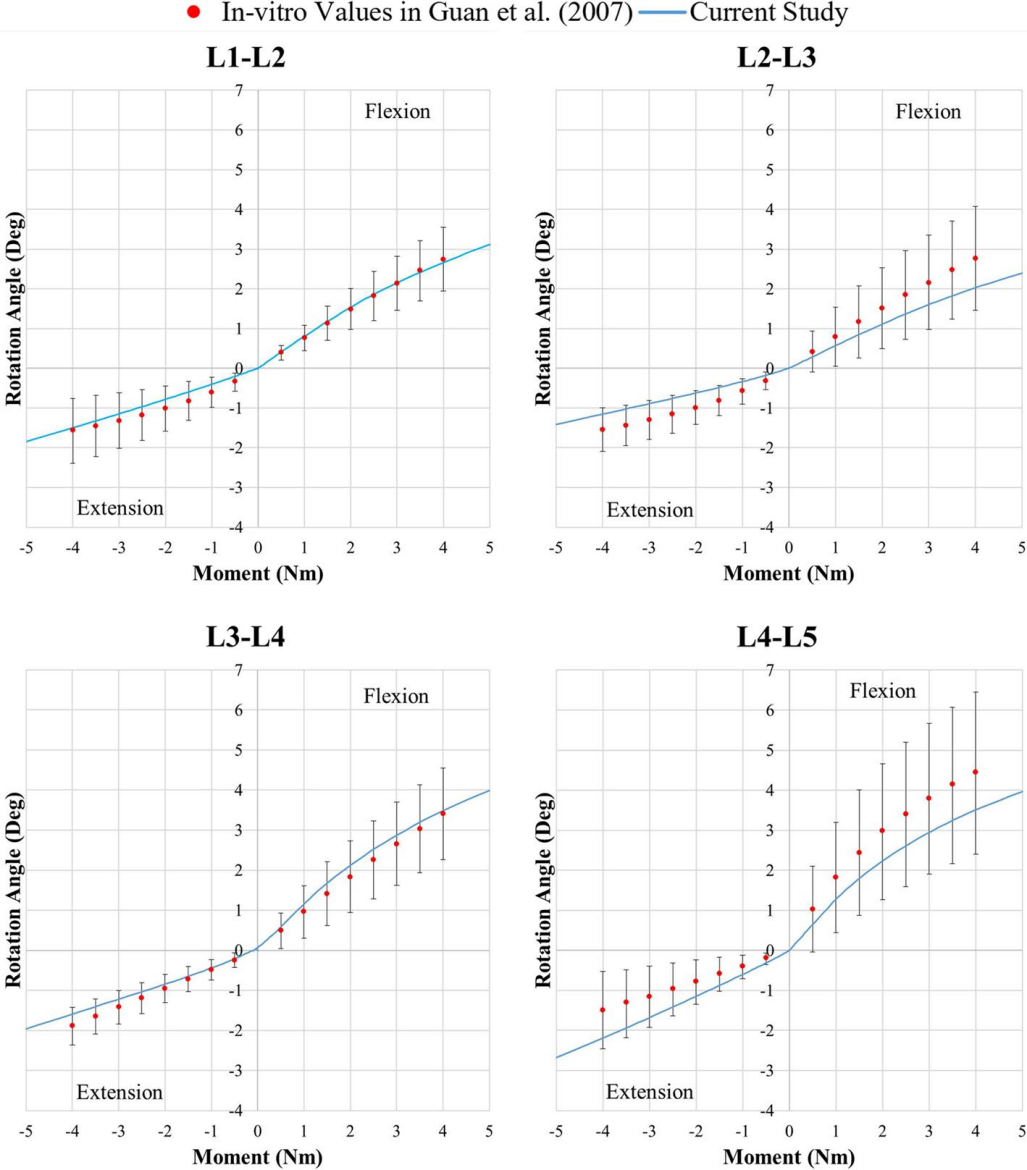
Comparison of moment–rotation responses at each spinal level under flexion–extension

In the literature, there are useful in vitro studies for the validation of FE models. For example, in their in vitro study, Guan et al. [27] investigated the hypothesis that L5–S1 behaves differently from the L1–L5 joints under pure moment load for flexion and extension and lateral bending motions. ROM and stiffness data were obtained using ten T12–S1 column specimens with ages ranging from 27 to 68 years with a load level of 4 Nm. In our verification of the flexion–extension movements of each segment, Guan et al.’s in vitro study [27] was taken as a reference in order to compare the ROMs at 4 Nm. All current results were in the ranges reported in that in vitro study. Moreover, some ROMs were very close to the mean values. Therefore, the flexion–extension ROMs of the FE model were consistent with those of Guan et al. (Fig. 1).

**Fig. 2:**
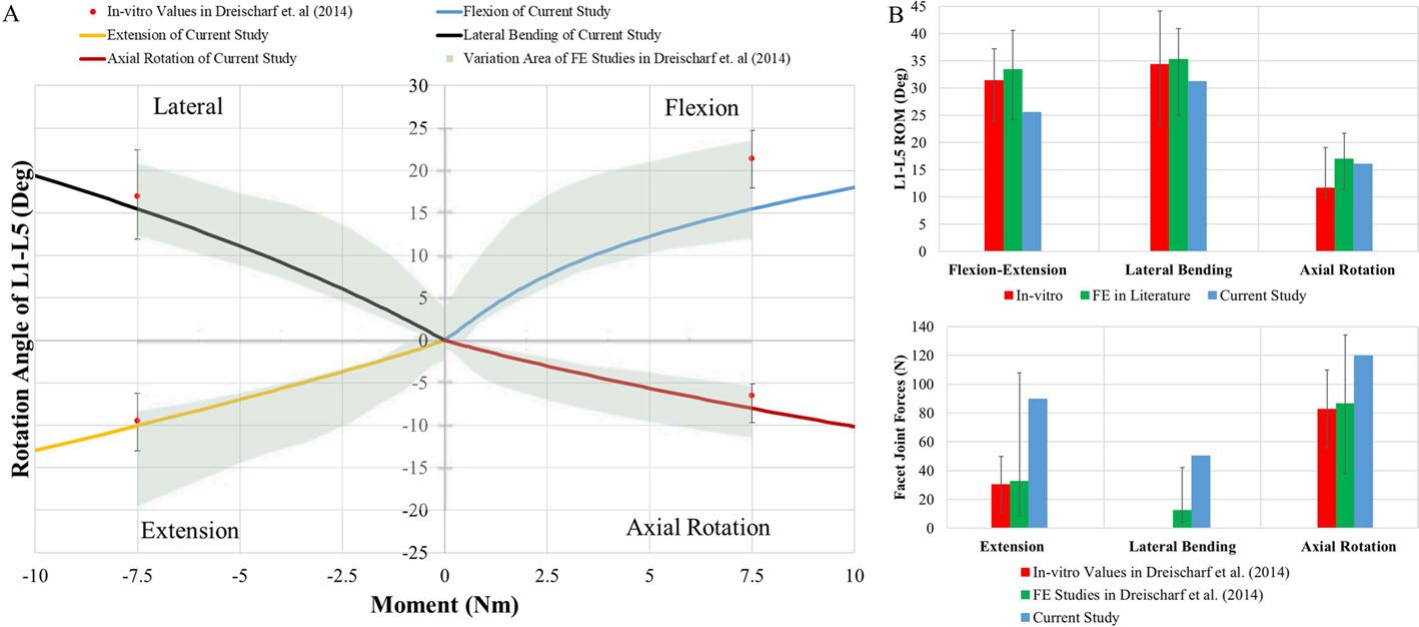
**A** Comparison of moment–rotation angle curves for biomechanics of L1–L5 lumbar spine. **B** Comparison of ROMs in the current FE model of L1–L5 lumbar spine with other FE models in the literature at 7.5 Nm. **C** Comparison of FJ loading in intact model of L1–L5 lumbar spine with in vitro and FE results in the literature

The study conducted by Dreischarf et al. [28] was also used to validate the current study. They investigated eight well-established FE models of the L1–L5 lumbar spine from different research centers by comparing in vitro and in vivo measurements for intervertebral rotations, disc pressures, and FJ loading under pure and combined loading modes. For the whole ROM of the L1–L5 lumbar spine model, a comparison was undertaken considering the study of Dreischarf et al. [28]. In Fig. 2A, the ROMs from the in vitro study and the area of variation formed by the external boundaries of the ROM curves of the eight FE models from Dreischarf et al.’s study are given; the ROMs of the in vitro study were used for validation [28, 29]. The variation area showed that the ROMs of the FE models could change in comparison to the in vitro values with different designs and material sets. All ROMs of the current study remained within the in vitro ROMs except for flexion motion. The current ROM for flexion motion was slightly below the in vitro ROM. This was caused by material properties and especially by the ligament set used in the current study. A comparison between the ROMs of the current study and the ROMs of the eight FE models is shown in Fig. 2B for a moment load of 7.5 Nm. The ROMs of the current study remained within the ROMs of the eight FE models for flexion–extension, lateral bending, and axial rotation motion. When the ROMs of the in vitro study and the FE models were considered, the current FE model showed close ROMs. Therefore, the current FE model was shown to be acceptable for ROM-based investigations. In Fig. 2C, the median FJ loadings of all spinal levels (L1–L5) of the eight FE models in extension, axial rotation, and lateral bending are given based on Dreischarf et al.’s study [28] according to measured FJ force with in vitro values in extension and axial rotation. Although the results of the current study were higher than the in vitro results for extension and axial rotation motion, they were still within the range of the median values of the FE models. For lateral bending, the result of the current study was slightly higher than the maximum value of the median of the FE models and there was no comparable in vitro result. In addition, the measuring of FJ loading is difficult and the values of FJ loading are variable due to structures and measurement methods that cause wide variation in results with FE models according to in vitro studies. Therefore, the results of the current study show some differences, but were deemed acceptable for investigating changes in FJ loading under different physiological conditions.

**Fig. 3:**
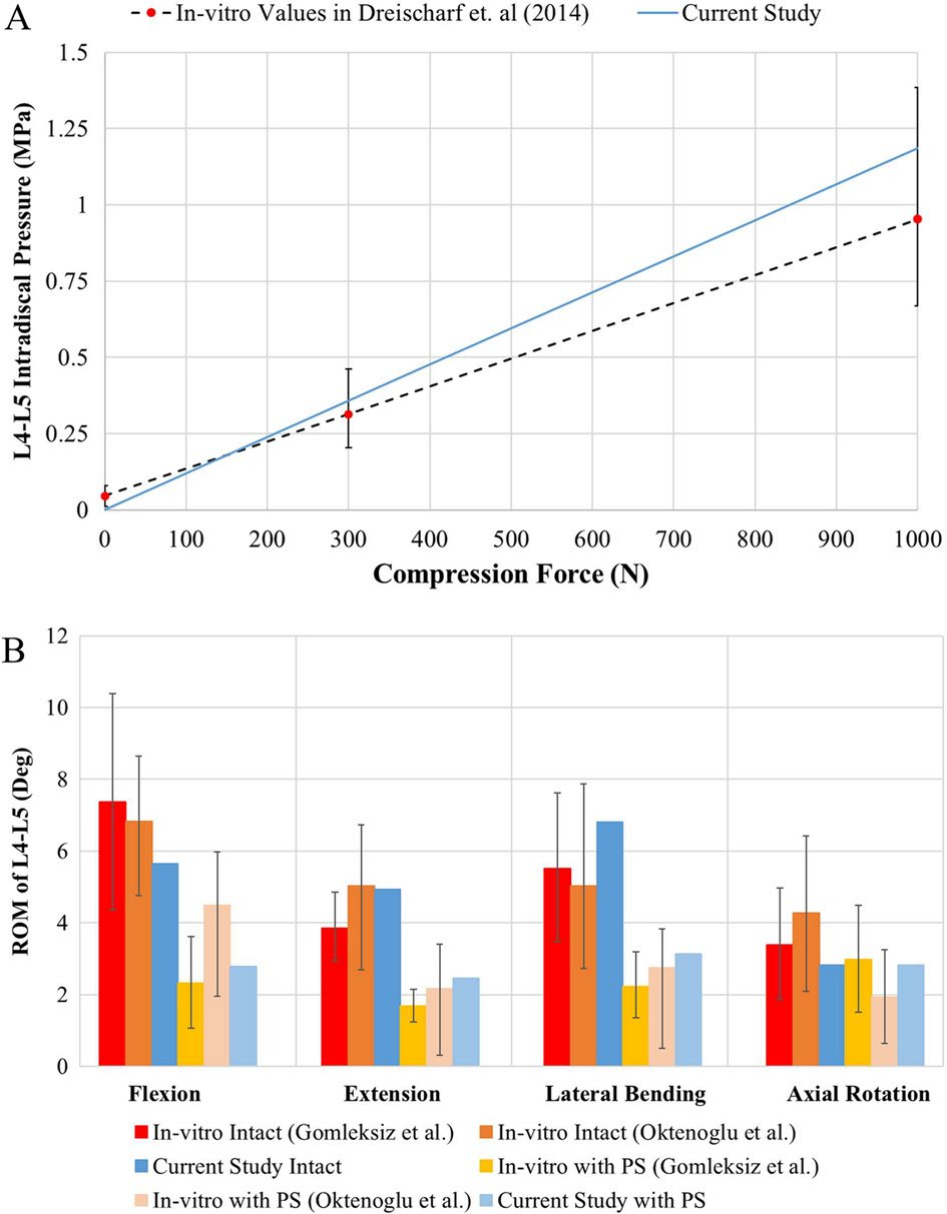
**A** Intradiscal pressure change of L4–L5 nucleus in the current FE model with compression force application. **B** Comparison of range of motion values of L4–L5 segment in intact and implanted models with two in vitro results (at loads of 10 Nm)

In addition, intradiscal pressure values for an intact L4–L5 nucleus considering the L4–L5 segments of 15 subjects were given in Dreischarf et al.’s in vitro study [28]. These pressure values were measured by applying compression forces at 0 N, 300 N, and 1000 N. Similarly, the intradiscal pressure of the L4–L5 nucleus was obtained by applying compression force from 0 to 1000 N on the L4–L5 nucleus in the current study. Thus, a straight line passing through the ranges of the in vitro results was obtained (Fig. 3A). It was accordingly seen that the results of the current study were consistent with the in vitro results for the intradiscal pressures of the L4–L5 segment.

The ROMs of the L4–L5 segment in an intact (INT) and an implanted (IMP) model were compared with the results of two in vitro studies in the literature, namely those of Gomleksiz et al. [11] and Oktenoglu et al. [14] (Fig. 3B). In Gomleksiz et al.’s study [11], L2–S1 cadaver specimens with implantation at the L4–L5 level were used and one of their models included PS fixation without a cage. The results of this model were considered for the comparison because no cage was used in the current study. In Oktenoglu et al.’s study [14], L2–S1 cadaver specimens with implantation at the L4–L5 level were also used and an intervertebral disc at the implanted L4–L5 level was partially removed. Although the intervertebral disc at the implanted L4–L5 level of the IMP model in the current study was not removed, the results of the experiment with rigid rod and rigid screws in Oktenoglu’s study [14] were considered for the comparison due to the negligible effect of the intervertebral disc at the implanted L4–L5 level. In general, it can be stated that the ROMs of the L4–L5 segment in the INT and IMP models without fusion were within the range of the results of the in vitro studies [11, 14].

### Range of motion

In the simulations, proper boundary conditions, described in more detail in the section below on boundary conditions, were applied for INT, IMP, and implanted with fusion (IMPF) models. The ROMs of the lumbar spine levels of all models for all physiological motions are listed in Table 1. In flexion, the ROMs of the L1–L2, L2–L3, and L3–L4 adjacent levels demonstrated 21%, 21%, and 12% increases for the IMP model, whereas 34%, 35%, and 32% increases occurred for the IMPF model, respectively. In extension, the ROMs of these adjacent levels showed 18%, 18%, and 29% increases for the IMP model, whereas 33%, 34%, and 55% increases were obtained for IMPF, respectively. In lateral bending, the ROMs of these adjacent levels reflected an increase of 21%, 24%, and 21% for the IMP model, whereas 34%, 39%, and 35% increases were gained for IMPF, respectively. In axial rotation motion, there were no significant changes in the ROMs of the IMP model at the adjacent levels. For all physiological motions, a gradual increase was observed for IMPF in the ROMs at the adjacent levels. At index level, approximately 42%, 41%, and 45% decreases in ROM were obtained without fusion; however, 81%, 77%, and 81% decreases in ROM were obtained with fusion for flexion, extension, and lateral bending motions, respectively. In axial rotation, there was no important change in the ROM at the index level without fusion; however, 87% decrease in ROM was obtained with fusion. Therefore, no significant effect was observed with posterior PS fixation in axial rotation without fusion. Furthermore, a decrease in ROM at the L4–L5 level was directly observed for all physiological motions after fusion. Before fusion, this decrease was observed for flexion, extension, and lateral bending motion.

**Table 1:** ROMs of intact FE model and implanted FE models with and without fusion for all motion segments

Motion	Model	L1–L2 (Deg)	L2–L3 (Deg)	L3–L4 (Deg)	L4–L5 (Deg)	Mom. (Nm)	L1–L5 stiffness (Nm/Deg)
Flexion	INT	4.08 (100%)	3.15 (100%)	4.97 (100%)	4.90 (100%)	7.5 (100%)	0.44 (100%)
	IMP	4.93 (121%)	3.82 (121%)	5.55 (112%)	2.86 (58%)	10.3 (137%)	0.60 (136%)
	IMPF	5.46 (134%)	4.26 (135%)	6.58 (132%)	0.94 (19%)	12.3 (164%)	0.71 (161%)
Extension	INT	2.61 (100%)	2.06 (100%)	2.58 (100%)	3.73 (100%)	7.5 (100%)	0.68 (100%)
	IMP	3.08 (118%)	2.44 (118%)	3.32 (129%)	2.22 (60%)	9.0 (120%)	0.81 (119%)
	IMPF	3.47 (133%)	2.76 (134%)	4.00 (155%)	0.87 (23%)	10.3 (137%)	0.93 (137%)
Lateral bending	INT	4.36 (100%)	2.85 (100%)	5.16 (100%)	5.46 (100%)	7.5 (100%)	0.42 (100%)
	IMP	5.27 (121%)	3.54 (124%)	6.23 (121%)	2.98 (55%)	9.5 (127%)	0.53 (126%)
	IMPF	5.84 (134%)	3.97 (139%)	6.95 (135%)	1.04 (19%)	10.8 (144%)	0.61 (145%)
Axial rotation	INT	2.10 (100%)	1.74 (100%)	2.08 (100%)	2.24 (100%)	7.5 (100%)	0.92 (100%)
	IMP	2.04 (97%)	1.69 (97%)	2.31 (111%)	2.14 (96%)	7.2 (96%)	0.88 (96%)
	IMPF	2.69 (128%)	2.26 (130%)	3.06 (147%)	0.30 (13%)	10.0 (133%)	1.20 (130%)

The percentages indicate the ROMs of all models normalized by the ROM of INT

### Facet joint loading

The FJ loading at each segment for all models in this study, obtained from the calculated contact forces between FJs in the FE models, is given in Table 2. The FJ loading in flexion was at a negligible level compared to the other physiological motions. FJ loading in flexion was not tabulated due to the negligible values of FJ loading during flexion motion [9, 30, 31]. In extension, the FJ loading of the L1–L2, L2–L3, and L3–L4 adjacent levels demonstrated 19%, 20%, and 35% increases for the IMP model, whereas 36%, 38%, and 54% increases were obtained for IMPF, respectively. Moreover, maximum increase occurred at the L3–L4 level for both models. In lateral bending, the FJ loading of these adjacent levels showed 22%, 25%, and 23% increases for the IMP model, whereas 43%, 51%, and 50% increases were gained for IMPF, respectively. In addition, there was an increase for all adjacent levels compared to the INT model, although no significant difference was observed among the increases at these levels. In axial rotation, the FJ loading of these adjacent levels indicated 33%, 33%, and 42% increases for IMPF, respectively. Moreover, there was a slight decrease at the L1–L2 and L2–L3 levels, whereas there was a slight increase at the L3–L4 level for IMP during axial rotation motion. The maximum increase in FJ loading was seen at the L3–L4 levels of both models in extension and axial rotation motion. At the implanted L4–L5 level of the IMPF and IMP models, FJ loading became zero in extension and lateral bending motion. However, in axial rotation motion, the FJ force remained nearly the same at the implanted L4–L5 level of the IMP model. It approached zero at the implanted L4–L5 level of the IMPF model in axial rotation motion.

**Table 2:** Comparison of predicted facet load of the INT, IMP, and IMPF models for all segments of the L1–L5 lumbar spine

	Extension				Lateral bending				Axial rotation			
Condition	L1–L2	L2–L3	L3–L4	L4–L5	L1–L2	L2–L3	L3–L4	L4–L5	L1–L2	L2–L3	L3–L4	L4–L5
INT	44.3 (100%)	95.2 (100%)	101 (100%)	117.6 (100%)	61.7 (100%)	63.8 (100%)	48.7 (100%)	26.4 (100%)	118.2 (100%)	133.4 (100%)	125.6 (100%)	101.3 (100%)
IMP	52.8 (119%)	114.5 (120%)	136.2 (135%)	0 (0%)	75.5 (122%)	79.7 (125%)	60.1 (123%)	0 (0%)	112.9 (96%)	126.9 (95%)	127.9 (102%)	96.8 (96%)
IMPF	60.2 (136%)	131.7 (138%)	155.7 (154%)	0 (0%)	88.5 (143%)	96 (151%)	73.2 (150%)	0 (0%)	157.1 (133%)	177 (133%)	178.1 (142%)	0 (0%)

Units of all values are Newton. Percentages indicate the ROM of all models normalized by the ROM of the intact model

### Posterior instrumentation stress

**Fig. 4:**
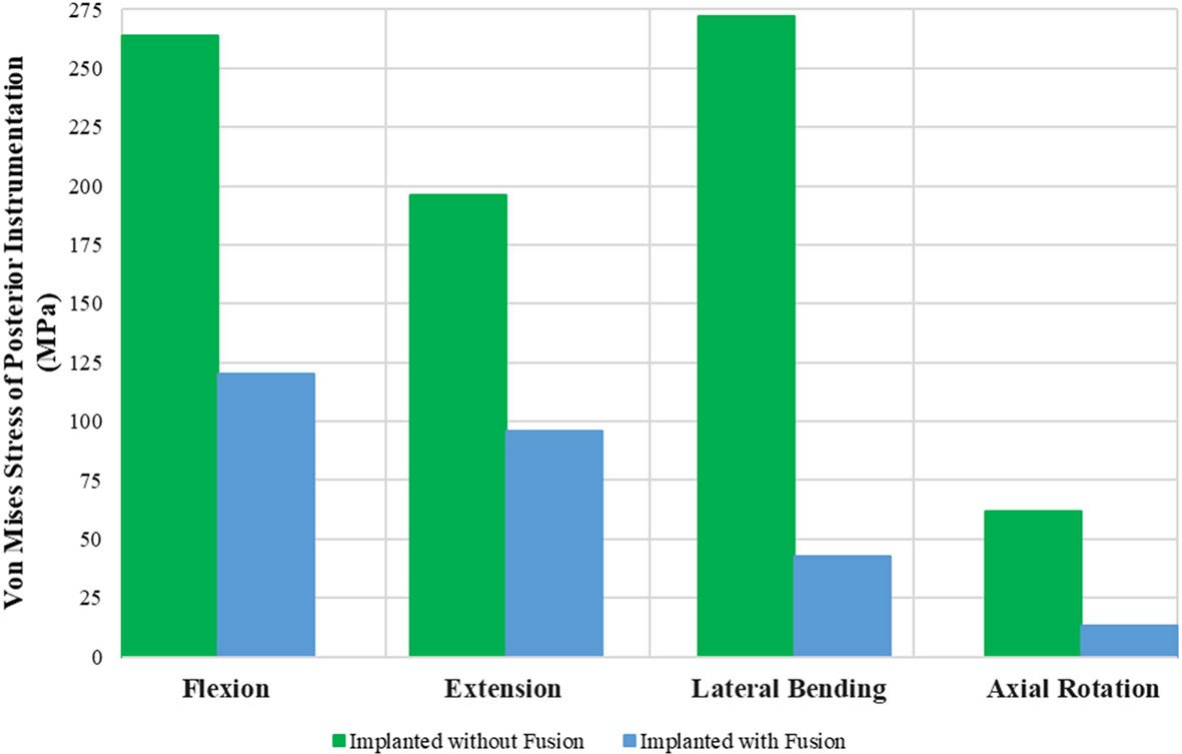
Maximum von Mises stresses on posterior instrumentation

**Fig. 5:**
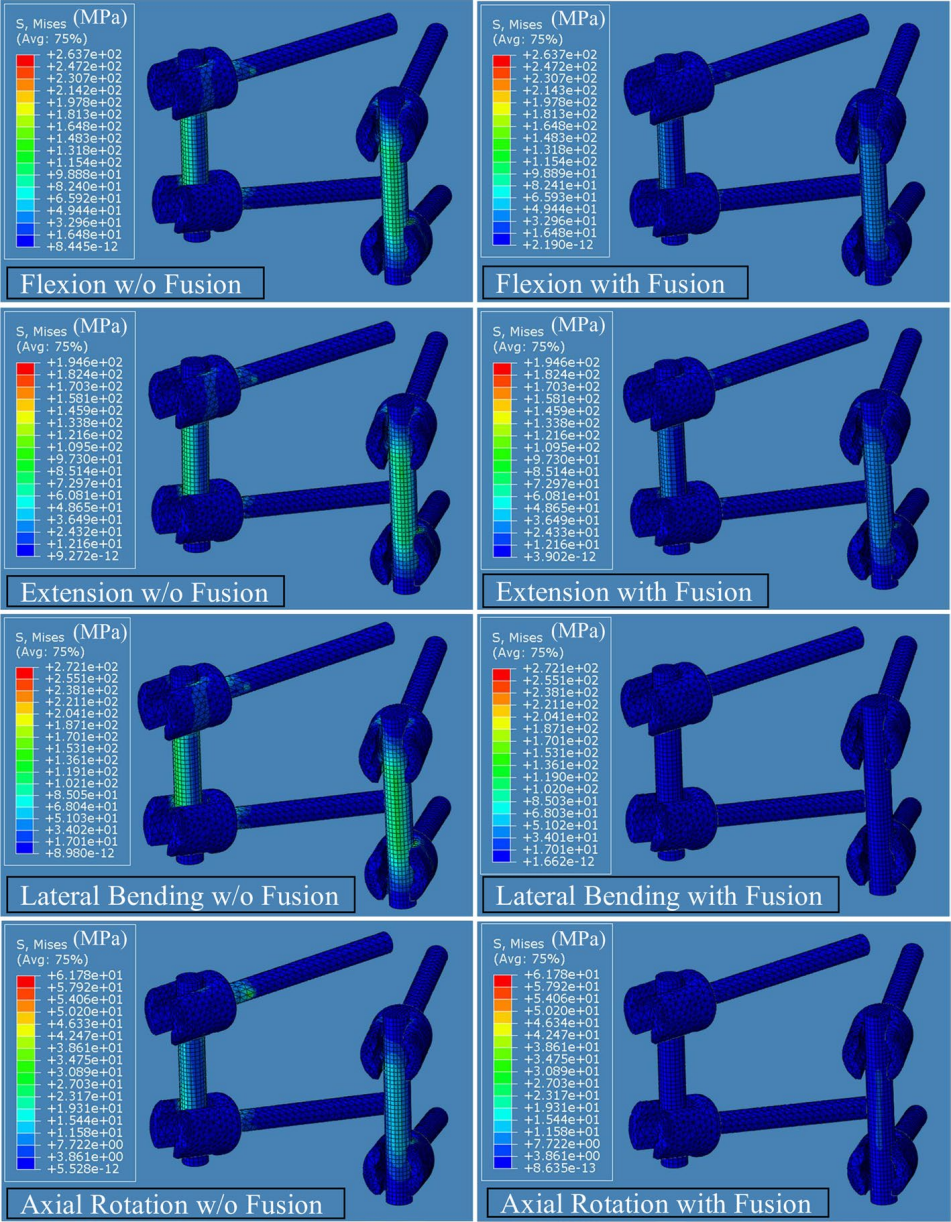
Stress nephogram of posterior instrumentations in flexion, extension, lateral bending, and axial rotation motion before and after fusion

The peak von Mises stresses on PI in the IMP and IMPF models under different physiological motion conditions are given in Fig. 4. Stress nephograms of PI in all physiological motions before and after fusion are given in Fig. 5. Before fusion, maximum stress of 272.1 MPa on PI occurred in lateral bending motion. After fusion, maximum stress of 120 MPa on PI occurred in flexion motion. With fusion, the greatest decreases in the stresses were seen in lateral bending and axial rotation motions. Moreover, maximum von Mises stresses of 272.1 MPa and 61.8 MPa decreased to 45.2 MPa and 13.3 MPa in lateral bending and axial rotation motion, respectively, while maximum von Mises stresses of 263.7 MPa and 196.4 MPa decreased to 120 MPa and 96.1 MPa in flexion and extension motion, respectively. Therefore, a general decrease in the von Mises stresses of the PI with fusion formation was observed. In addition, maximum stress occurred in the middle field of the rods.

## Discussion

It has been shown that FE analysis has more advantages than experimental methods. FE studies are also preferred to experimental studies because of difficulties in experimental studies in adapting to the changes in the FJ force or disc stresses due to different implantation and motion conditions [30]. Recently, FE studies have focused on the hypothesis that lumbar pedicle fixation and interbody fusion cause an increase in ASD due to the increment of FJ force and the ROM at adjacent segments [18–21]. Furthermore, different alternative non-fusion methods have been developed to eliminate the possible drawbacks of fusion formed with PS fixation, such as ASD, screw loosening, and rod and screw breakage [21–24]. However, the new alternative methods also have some drawbacks [11, 25, 26]. The common feature of all these methods is that they use posterior PS fixation. Therefore, the most important goal is to achieve a better understanding of the effects of posterior PS fixation on the biomechanics of the lumbar spine with and without fusion. ROM and FJ force are main indicators for the performance of spinal fusion [31]. In this study, these main indicators and PI stresses were investigated under the influence of different physiological motions.

In the study of Chen et al. [9], comparing FE models of the intact spine, the Awesome Dynamic Rod System was implanted at L4–L5, a traditional rigid rod system was implanted at L4–L5 along with an interbody cage (FUS), and the Awesome Dynamic Rod System was implanted at L4–L5 along with an interbody cage. The authors aimed to demonstrate the effects of dynamic rod systems on adjacent levels of fusion segments in terms of ROMs, disc stresses, and FJ forces. Similarly to that study, it was observed in the present work that segment stiffness values usually increased with instrumentation. In addition, there was an increase in the ROMs of the IMPF model in the current study and the FUS model in Chen et al.’s study [9] at the L1–L2, L2–L3, and L3–L4 levels with all physiological motions compared to INT. The ROMs at L4–L5 in the FUS model were 0.76°, 0.55°, 0.94°, and 1.84° with flexion, extension, lateral bending, and axial rotation motion, respectively, while those of the IMPF model in the current study were 0.94°, 0.87°, 1.04°, and 0.30° with flexion, extension, lateral bending, and axial rotation motion, respectively. Thus, the ROMs at the L4–L5 level in the previous FUS model and the current IMPF model are close for flexion and lateral bending motions. Li et al. [10] analyzed the following five fixation models: unilateral pedicle screw fixation, graft fusion with unilateral pedicle screw fixation, bilateral pedicle screw fixation (BPS), graft fusion with bilateral pedicle screw fixation (F-BPS), and removal of posterior instrumentation after graft fusion. They aimed to compare stability between unilateral pedicle screw fixation and bilateral pedicle screw fixation before and after graft fusion. As in the F-BPS model of Li et al.’s study [10], the ROMs at the L4–L5 level in the IMPF model in the current study were close and under 1° for all motion conditions.

Another study conducted by Kim et al. [30] investigated the relationship between the position of an inserted pedicle screw and the related facet contact force or intradiscal pressure. They used four L4–L5 fusion models according to the positions of pedicle screws in L4 of the L4–L5 lumbar fusion. One of these models, referred to as the facet violation (FV) model, entailed the violation of both L3–L4 superior FJs by pedicle screws. The FV model was comparable to the implanted models of the current study, considering FJ loading in extension and axial rotation motion. While 166.7% and 132.1% increases were obtained in FJ force at the L3–L4 level in the FV model with extension and axial rotation motion, respectively, compared to that study’s own INT model, 54% and 42% increases were found in FJ force at the L3–L4 level in the IMPF of the current study in extension and axial rotation motion, respectively. There was a significant difference between the increases in FJ loading among these studies in the literature. These great differences in FJ force increases might originate from the PS positioning in the model, as mentioned by Kim et al. [30]. Nevertheless, it can be concluded from the studies in the literature that posterior PS fixation causes an increase in FJ force at adjacent levels [9, 30]. In this study, there was a slight decrease in FJ force at the L1–L2 and L2–L3 levels, whereas there was a slight increase in FJ force at L3–L4 for IMP during axial rotation motion. This condition could have arisen from the partial removal of FJs and capsular ligaments due to the applied surgical procedure. In Chen et al.’s study [9], FJ force at the L4–L5 level in the FUS model decreased to zero, as in the IMPF model in the current study, and especially with extension and lateral bending motion. In addition, FJ force approached zero at the L4–L5 level of IMPF with axial rotation motion while it showed an 86% decrease at the L4–L5 level of the FUS model with axial rotation motion.

In this study, the peak von Mises stresses on PI for different physiological motion conditions were investigated. Before fusion, maximum stress of 272.1 MPa on PI occurred with lateral bending motion. After fusion, maximum stress of 119.8 MPa on PI occurred with flexion motion. With fusion formation, 84% and 78% decreases were seen with lateral bending and axial rotation motion, respectively. The von Mises stresses in PI of the BPS and F-BPS models employed in Li et al.’s study [10] were generally less than those of IMPF and IMP in the current study. However, the maximum stress decreases of PI were seen in both studies for lateral bending and axial rotation motion after fusion. Moreover, the peak von Mises stresses on PSs under different physiological motion conditions were also investigated and maximum von Mises stresses were seen on the neck field of the screws in this study [32, 33]. It was then seen that von Mises stresses on PI decreased for all physiological motions after fusion. Therefore, the stresses on PI before fusion could cause the failure of the spinal fixation.

### Limitations

There were some limitations of the current study. The PS model was formed by ignoring threads and connections between screws, while the bone and rods were made rigidly. Therefore, screw loosening effects were not considered in this study. The lumbar spine models in this study could only be used for patients of a specific age range and gender because they were based on computed tomography (CT) data from only one subject. In this study, no follower load was applied. There are many studies which have different follower load values, and the instrumentation stresses and FJ loads will change according to different follower load values. Thus, if a follower load had been applied, the values of instrumentation stress and FJ load would have been greater than the values obtained in this study without any follower load. In addition, stress distribution in ligaments was not considered because ligaments were modeled as tension-only two-node truss elements. If stress distribution in ligaments is to be considered, they should be reformed by solid elements. In this study, bony tissues and implants were modeled using linear-elastic material properties [4]. It was appropriate to use linear-elastic material properties to simulate pre-yield conditions since this study did not consider post-yield conditions. Nevertheless, linear-elastic material properties could be used when bone tissues and implants are subjected to minor deformations. Nonlinear-elastic material properties should be used to anticipate the failure and yielding processes of bone tissues and implants in high-strain energy situations. To obtain a more precise analysis of the stress and breakage risk of PI, further fatigue analyses should be performed by considering patient-specific anatomical and material properties together with the types and positions of implants.

## Conclusions

In this study, the amount of decrease in ROM restrictions at the implanted L4–L5 level after fusion and FJ force increments at adjacent levels were determined. Posterior PS fixation caused a significant increase in FJ loading at the L3–L4 adjacent level, especially with extension and lateral bending motion. This FJ loading increment at the adjacent level could be accepted as support for the hypothesis that ROM restrictions and FJ force increments cause ASD. In addition, high peak von Mises stresses on PI occurred with lateral bending and flexion motion before fusion. Higher stresses would then have increased the failure risk of PI under subsequent loadings due to physiological motions of the lumbar spine. Before successive clinical experiments on lumbar spine biomechanics, this model could help provide prior knowledge for future studies. Moreover, the stress on PI before fusion could be decreased by alternative methods such as non-fusion dynamic fixation systems or anterior support applications. The aim of this FE study has been to determine how a posterior PS fixation system affects the biomechanics of the lumbar spine before and after fusion. With this developed intact model, a fundamental tool has been created for future studies to explore topics such as multiple-segment PS fixation, artificial disc implantation, and different surgical techniques.

## Methods

### Intact model

**Fig. 6:**
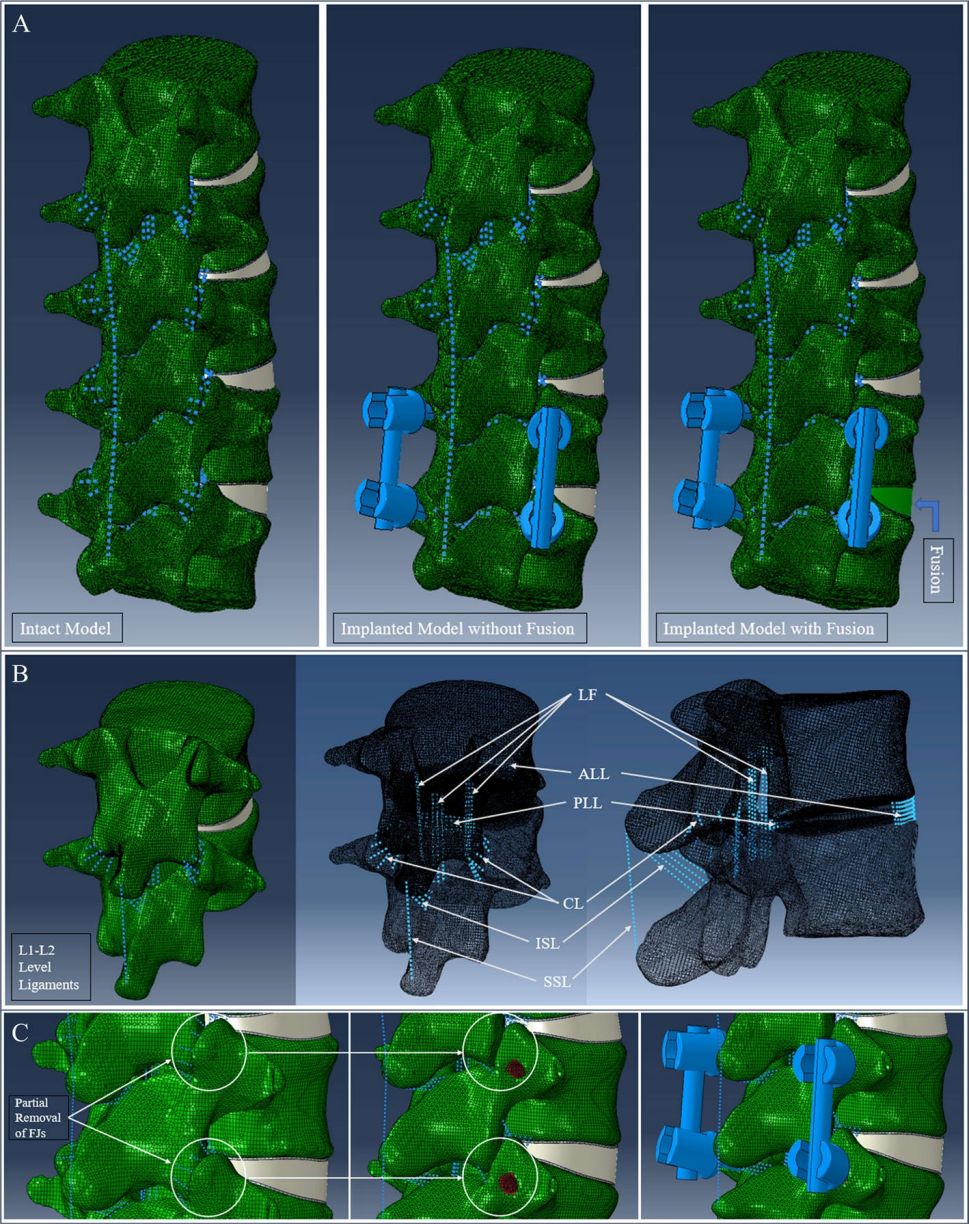
**A** Intact and implanted lumbar spine FE model with and without fusion. **B** Types and locations of ligaments shown on the L1–L2 segment. **C** Partial removal of FJs at the implanted L4–L5 level

CT image data of a healthy full human spine with anatomically intact structure were taken from the TOBB ETU data and processed in 3D Slicer (https://www.slicer.org/) to obtain a proper model in.stl format [34]. A lumbar vertebral section was separated from the model in CATIA (Version 5.0; Dassault System’s, France), a 3D CAD modeling program. During generation of cortical shell sections, SpaceClaim (2019; ANSYS, USA) was used for surface offsetting, converting.stl format models into.iges and.stp format files. Moreover, lumbar vertebral parts from L1 to L5 and the vertebral discs between them were created as 3D solid models by making the required modifications. BOLT (Version 2.0; Csimsoft, USA) was used for the meshing of vertebrae, including cortical shell and cancellous bone, and then the analysis model was generated in ABAQUS (Version 2017; Abaqus, USA) with all components such as vertebrae, discs, ligaments, and endplates. The L1–L5 lumbar vertebrae model in this study had a 42° lordotic angle, compatible with the mean lordotic angles of the L1–L5 lumbar segments of adolescents and adults in the literature [35]. The dimensions of the 3D model of the vertebrae were controlled according to the overall vertebral dimensions in this age range from studies in the literature [36–38]. The thickness of cortical bone was considered as 0.5 mm, as in the overall value of studies in the literature [36, 39–41], while 12,000 MPa and 0.3, also values widely used in the literature, were taken as the elastic modulus and Poisson’s ratio of the cortical bone, respectively. The inner side of the vertebra was assigned the widely used material properties of cancellous bone, which are 100 MPa elastic modulus and 0.2 Poisson’s ratio [42]. Considering the studies in the literature, the annulus fibrosus was represented in this study with a solid ground matrix and six membrane layers having mean thickness of 0.12 mm with uniformly spaced reinforcing bars [43–45]. For the ground matrix and six membrane layers of the annulus section, the Mooney–Rivlin hyperelastic material property was assigned. Moreover, reinforcing bars, representing fibers in the annulus fibrosus, were modeled by rebar elements (beam-like elements) in ABAQUS [46]. Mechanical properties of the nucleus pulposus were defined as a nearly incompressible material [47]. Endplate parts of the intervertebral discs were created by offsetting the elements on the upper and bottom surfaces of the annulus and nucleus, and the endplate thickness was taken as 0.5 mm in light of studies in the literature [39, 41, 48]. In this study, six ligament types, namely the anterior longitudinal (ALL), posterior longitudinal (PLL), ligamentum flavum (LF), interspinous (ISL), supraspinous (SSL), and capsular (CL) ligaments, were considered (Fig. 6B). The intertransverse ligament (ITL) was ignored because the reference studies excluded ITL data. Moreover, nonlinear stiffness data of ligaments, obtained from the test data of Shirazi-Adl et al. [49] and Schmidt et al. [50] and as indicated in Naserkhaki et al.’s study [51], were implemented for each ligament type separately. Whereas the stiffness values of ligaments from among the test data of Shirazi-Adl et al. [49] were used for the ALL and PLL, those of Schmidt et al. [50] were used for the other ligaments. All ligaments were modeled without using compression truss elements in ABAQUS. For FJs, the facet modeling method of exponential force transfer between the nodes according to 0.1-mm initial gap size was used [39]. In this contact definition, the contact pressure on the FJ surface varies exponentially according to the distance between the joint surfaces. The maximum contact pressure that may occur between the surfaces was defined to be equal to the modulus of elasticity of the surrounding bone. Moreover, the friction coefficient for the contact between the inferior and superior joint surfaces was taken as 0.1 [48]. The related master and slave surfaces between the vertebrae were then determined for each FJ in order to make interaction contacts in ABAQUS. In the study conducted by Yamamoto et al. [52], the authors applied 50 N, 100 N, and 150 N pre-loads and stated that pre-loading did not statistically affect the ROMs of the lumbar spine. However, Rolmann et al. [53] performed a similar study and stated that applying a follower load did not affect the ROM significantly for lateral bending and flexion–extension, but only for axial rotation during an application of 7.5 Nm moment. Therefore, a follower load was not applied in our study. Considering all of the points mentioned above, the L1–L5 FE lumbar spine model was generated with the material properties listed in Table 3.

**Table 3:** Material properties and element types of lumbar FE model components

FE sections	Young’s modulus (MPa)	Poisson’s ratio	Elements (FEM)	References
Cortical bone	12,000	0.3	S3 triangular shell elements	Kurutz [42]
Cancellous bone	100	0.2	C3D8R hexahedral elements	
Endplate	23.8	0.4	C3D8R hexahedral elements	
Nucleus pulposus	1	0.499	C3D8R hexahedral elements	Galbusera et al. [47]
Annulus ground substance	Mooney–Rivlin, C10 = 0.13, C01 = 0.03, D = 0.6		C3D8R hexahedral elements	Wang et al. [54]
Annulus fibrosus layers	Mooney–Rivlin, C10 = 0.13, C01 = 0.03, D = 0.6		M3D4R quadrilateral elements	
Fibers of layer 1	550	0.45	Rebar	Tsouknidas et al. [45]
Fibers of layer 2	495	0.45	Rebar	
Fibers of layer 3	440	0.45	Rebar	
Fibers of layer 4	420	0.45	Rebar	
Fibers of layer 5	385	0.45	Rebar	
Fibers of layer 6	360	0.45	Rebar	
Ligaments	Nonlinear stress–strain curves	–	Connectors	Naserkhaki et al. [51]
Pedicle screws and rods	110,000	0.3	C3D8R-C3D10 hexahedral elements–quadrilateral elements	Kang et al. [5]

### Boundary conditions

**Fig. 7:**
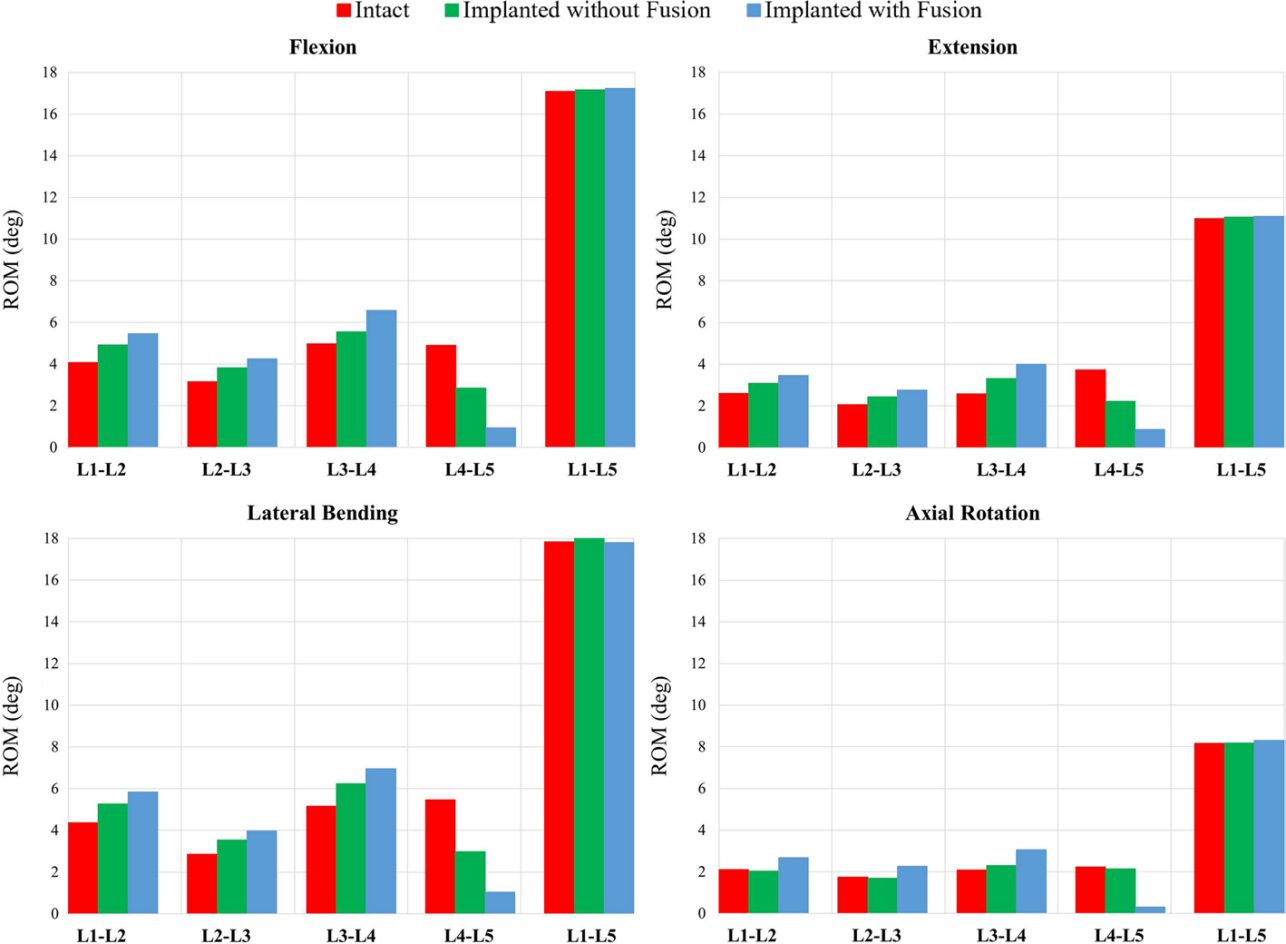
ROMs of intact model, implanted model without fusion, and implanted model with fusion for all physiological motions

In the FE model, two main reference points were determined over the center of the superior surface of the L1 vertebral body and below the center of the inferior surface of the L5 vertebral body. Moment loads were applied to the main reference point connected by coupling method with the superior surface of the L1 vertebral body, whereas the other main reference point related to the inferior surface of the L5 vertebral body was fixed with six degrees of freedom. The moment loads were then applied in the directions of flexion, extension, lateral bending, and axial rotation motions separately. Pure bending moment loads of up to 10 Nm were applied to the superior reference point of the FE model in all physiological loading directions. ROMs for each level of the L1–L5 lumbar spine were obtained for a comparison with the results of the studies in the literature by obtaining the biomechanical results of moment loads of up to 10 Nm [11, 14, 27, 28]. For analyses of implanted models, a hybrid method was used. This method is particularly recommended for investigations of adjacent level effects [55]. Compared to other methods, it is an appropriate method for evaluating adjacent level effects, using a familiar methodology and providing high-quality and laboratory-independent results for both fusion and non-fusion devices [55]. For application of this hybrid testing protocol, the intact load control values were obtained with pure moment load of 7.5 Nm in this study [8]. Unconstrained pure moment was then applied to implanted models until the total ROM of the implanted model was equal to the ROM of the intact load case [17, 43, 55]. Therefore, pure unconstrained moments were applied to ensure that the total ROM of the implanted models would be equal to 17° in flexion, 11° in extension, 8° in axial, and 18° in lateral bending (Fig. 7).

### Implanted model

A lumbar spine model with posterior PS instrumentation at the L4–L5 level was created to investigate surgical issues. Moreover, PSs were located on lumbar vertebrae in the FE model considering surgical operation methods. The PS model, with a mean outer diameter of 6.5 mm and a length of 45 mm, was obtained from Normmed Medical and Machinery Industry (Ankara, Turkey). It was then modified by simplification to achieve a proper mesh set for analysis. For this simplification, the body of the PS was modeled as a cylinder with diameter of 5 mm and length of 45 mm, assuming the diameter of the thread part of the PS as 5 mm [5]. In addition, two straight cylinder rods with diameter of 6 mm and length of 45 mm were modeled for the L4–L5 level. A rigid connection was then formed among PSs and rods by using the ‘Tie Contact’ feature in ABAQUS. Similarly, connections between screws and vertebrae were made with the same feature. The screw, heads, and rods were considered to be made of Ti–6Al–4V [5] with material properties as given in Table 3. Furthermore, the vertebrae related to the implanted L4–L5 level were modified with partial removal of FJs according to the surgical procedure as described in the surgical operation model modification section below. The L1–L5 FE lumbar spine model with posterior PS instrumentation at the L4–L5 level was formed and the related boundary conditions were applied. The other FE models, namely the IMPF and IMP, were formed by modifying the INT model. Whereas IMP represented the lumbar spine before fusion, IMPF represented the lumbar spine after fusion. After fusion, the disc nucleus loses its gel-like structure and hydrostatic properties due to degenerative processes [56]. Therefore, fusion was modeled in IMPF for this disc by assigning cortical bone material to discs and endplates at the implanted L4–L5 segment for fusion [57]. The disc representing the conditions before fusion was modeled as a healthy disc with the material properties given in Table 3. In short, the material properties of the intact disc and endplate were used in the IMP model. Thus, models were prepared in order to investigate the influence of the posterior PS fixation system on the biomechanics of the lumbar spine with and without fusion (Fig. 6).

### Surgical operation model modification

Implanted vertebrae were modified for the integration of PSs by removing the edge part of the superior articular facet in a way appropriate for the selected surgical procedure (Fig. 6C). A flat surface was obtained by removing the edge part of the superior articular facet. Then, by taking the transverse process as a reference, PS thread cavities on the vertebrae were formed to properly locate PSs. The depths of these cavities were opened, ensuring that the edges of the PSs were close to the cortical sections of vertebral bodies. After positioning of PSs, the modified 3D vertebral models were meshed. In addition, partial removal of the FJs resulted in removal of some connectors for the capsular ligaments on the posterior side. Moreover, all ligaments at the implanted level were rearranged by creating new truss elements passing through nodes close to the previous nodes of ligaments.

### Mesh convergence and validation

Three mesh densities (coarse mesh set: 261,829 elements/392,557 nodes; normal mesh set: 461,858 elements/634,885 nodes; finest mesh set: 1,319,720 elements/991,228 nodes) were prepared to evaluate the components in the intact model by convergence test. The density of the mesh was determined by convergence studies while ensuring that the coarsening of the mesh would not disturb the stress field by more than 2% [58]. The von Mises stress values of cortical bone, cancellous bone, endplate, nucleus pulposus, and annulus sections were compared by using these different mesh sets. The differences of von Mises stress between the normal mesh set and finest mesh set were less than 2% for all the tissues in the model. This model was validated according to the trend validation concept [59]. The behavior of the model was evaluated with in vitro studies with respect to the indication of trends in different loading conditions [27, 28]. The mesh quality was selected according to the literature data, considering element type and characteristics [60]. The whole model comprised linear hexahedral elements, with an average element side length amounting to 1 mm. Approximately 90% of the elements presented an aspect ratio between 1 and 2.5 and their average aspect ratio was 1.5. The elements had average element side length up to 1 mm, whereas the maximum element side length did not exceed 2 mm. Reduced integration elements were used in the model to prevent element shear locking, which may particularly take place in the simulation of viscoelastic tissue [56]. The final model consisted of 634,885 nodes and 461,858 hexahedral elements.

## Data Availability

All data generated or analyzed during this study have been included in this published article.

## Abbreviations

PS: Pedicle screw
FE: Finite element
FJ: Facet joint
PI: Posterior instrumentation
ASD: Adjacent segment disease
ROM: Range of motion
INT: Intact
IMP: Implanted without fusion
IMPF: Implanted with fusion
FUS: A traditional rigid rod system implanted at L4–L5 along with an interbody cage
BPS: Bilateral pedicle screw fixation
F-BPS: Graft fusion with bilateral pedicle screw fixation
FV: Facet violation
CT: Computed tomography
3D: Three-dimension
